# d-Allulose 3-epimerase of *Bacillus* sp. origin manifests profuse heat‐stability and noteworthy potential of d-fructose epimerization

**DOI:** 10.1186/s12934-021-01550-1

**Published:** 2021-03-04

**Authors:** Satya Narayan Patel, Girija Kaushal, Sudhir P. Singh

**Affiliations:** Center of Innovative and Applied Bioprocessing, S.A.S. Nagar, Sector-81 (Knowledge City), 140306 Mohali, India

**Keywords:** d-Allulose, d-Allulose 3-epimerase, *Bacillus* sp., Thermostability, Turnover number

## Abstract

**Background:**

d-Allulose is an ultra-low calorie sugar of multifarious health benefits, including anti-diabetic and anti-obesity potential. d-Allulose 3-epimerase family enzymes catalyze biosynthesis of d-allulose *via* epimerization of d-fructose.

**Results:**

A novel d-allulose 3-epimerase (DaeB) was cloned from a plant probiotic strain, *Bacillus* sp. KCTC 13219, and expressed in *Bacillus subtilis* cells. The purified protein exhibited substantial epimerization activity in a broad pH spectrum, 6.0–11.0. DaeB was able to catalyze d-fructose to d-allulose bioconversion at the temperature range of 35 °C to 70 °C, exhibiting at least 50 % activity. It displaced excessive heat stability, with the half-life of 25 days at 50 °C, and high turnover number (*k*_cat_ 367 s^− 1^). The coupling of DaeB treatment and yeast fermentation of 700 g L^− 1^ d-fructose solution yielded approximately 200 g L^− 1^ d-allulose, and 214 g L^− 1^ ethanol.

**Conclusions:**

The novel d-allulose 3-epimerase of *Bacillus* sp. origin discerned a high magnitude of heat stability along with exorbitant epimerization ability. This biocatalyst has enormous potential for the large-scale production of d-allulose.

## Background

d-Allulose or d-psicose or d-*ribo*-2-hexulose is a non-fermentable ketohexose sugar of low-calorie and rare occurrence in nature. Its demand is extensively increasing in food, nutraceutical, and pharmaceutical industries owing to its hypoglycemic, hypolipidemic, antioxidant, antiobesity, neuroprotective, and rheological properties, with a pleasant taste [1–6]. Recent studies have evidenced the role of d-allulose in the modulation of gut microfloral and probiotic community profile [7]. Its consumption ameliorates insulin resistance, offering prevention of diabetes [8, 9]. Furthermore, its beneficial effects in regulating the metabolic disturbance and brain function in prediabetic condition have recently been demonstrated [10]. The safety of this molecule had been well established by United States Food and Drug Administration (FDA), bestowing generally regarded as safe (GRAS) status (GRN Nos. 400, 498, 624, 647, and 693) for its use in food products as a nutritive functional ingredient. With all these properties, d-allulose is a preferred sweet functional ingredient in beverages, and other food products such as sauce, toppings, bakery products, yogurt, ice cream, etc. [11]. In recent years, it has garnered great attention in the markets of Japan, Korea, USA, and UK.

The chemical route of d-fructose to d-allulose transformation had been achieved by employing several chemo-catalysts, e.g., 1,2:4,5-di-O-isopropylidene-β-d-fructopyranose [12], molybdate ion [13], and pyridine [14]. Recently, natural polyamine spermine was used for the conversion of d-glucose to d-fructose, and d-allulose was obtained as a by-product [15]. However, chemical processing for d-allulose synthesis is considered to be complex and expensive. Most importantly, its chemical synthesis involves the generation of hazardous by-products, and therefore, is not environment friendly [16].

The epimerization of d-fructose at the C-3 position, catalyzed by a ketose 3-epimerase, is the prevailing biological approach for d-allulose production worldwide. Mainly four types of ketose 3-epimerases have been characterized from different bacterial sources: d-tagatose 3-epimerase, d-fructose 3-epimerase, L-ribulose 3-epimerase, and d-allulose 3-epimerase. These four enzymes display relatively higher substrate specificity towards d-tagatose, d-fructose, L-ribulose, and d-allulose, respectively. Nonetheless, they have the same characteristics of catalyzing d-fructose to d-allulose conversion [11, 17, 18]. The first d-allulose 3-epimerase (DAEase) was reported from *Agrobacterium tumefaciens* [19]. Subsequently, it has been characterized from various bacterial strains, e.g., *Clostridium cellulolyticum* H10 [20], *Ruminococcus* sp. [21], *Clostridium scindens* 35704 [22], *Clostridium* sp. [23], *Desmospora* sp. 8437 [24], *Clostridium bolteae* [25], *Dorea* sp. CAG317 [26], *Treponema primitia* ZAS1 [27], *Flavonifractor plautii* [28], *Arthrobacter globiformis* [29], *Agrobacterium* sp. ATCC 31749 [30], *Paenibacillus senegalensis* [31], *Staphylococcus aureus* [32], and *Rhodopirellula baltica* [33]. In the literature, thermal stability has been discussed as a critical factor in the enzymatic production of d-allulose at the industrial scale. Thermal tolerant variants of DAEase had been developed by following the protein engineering approaches [32–36]. Recently, our group characterized a novel thermotolerant DAEase by exploring the extreme temperature ecological niche [37].

Many of the DAEases were reported from the strains with the historical background of pathogenesis, e.g., *F. plautii* [38], *S. aureus* [39], *Desmospora* sp. [40], *Clostridium bolteae* [41], *T. primitia* [42], and *Rhodopirellula baltica* [43]. Some strains are pathogenic to plants, e.g., *P. cichorii*, and *Agrobacterium tumefaciens*. It is desirable to pick out DAEase from the non-pathogenic and safe microbial sources, keeping in mind their intensive implementation in food-grade processes.

The present investigation identified a novel DAEase of *Bacillus* sp. origin. This genus has lesser pathogenic potential and noteworthy microbiological uses in various industries [44]. *Bacillus* sp. KCTC 13219 is a versatile plant probiotic strain capable of inducing systemic resistance in plants, and thus crucial in eco-friendly agricultural applications [45]. The present study determined DAEase from *Bacillus* sp. strain KCTC 13219 to be an excessively thermostable enzyme with the high turnover number, and thus, suitable for industrial production of d-allulose.

## Results

### Gene identification and sequence analysis

**Fig. 1:**
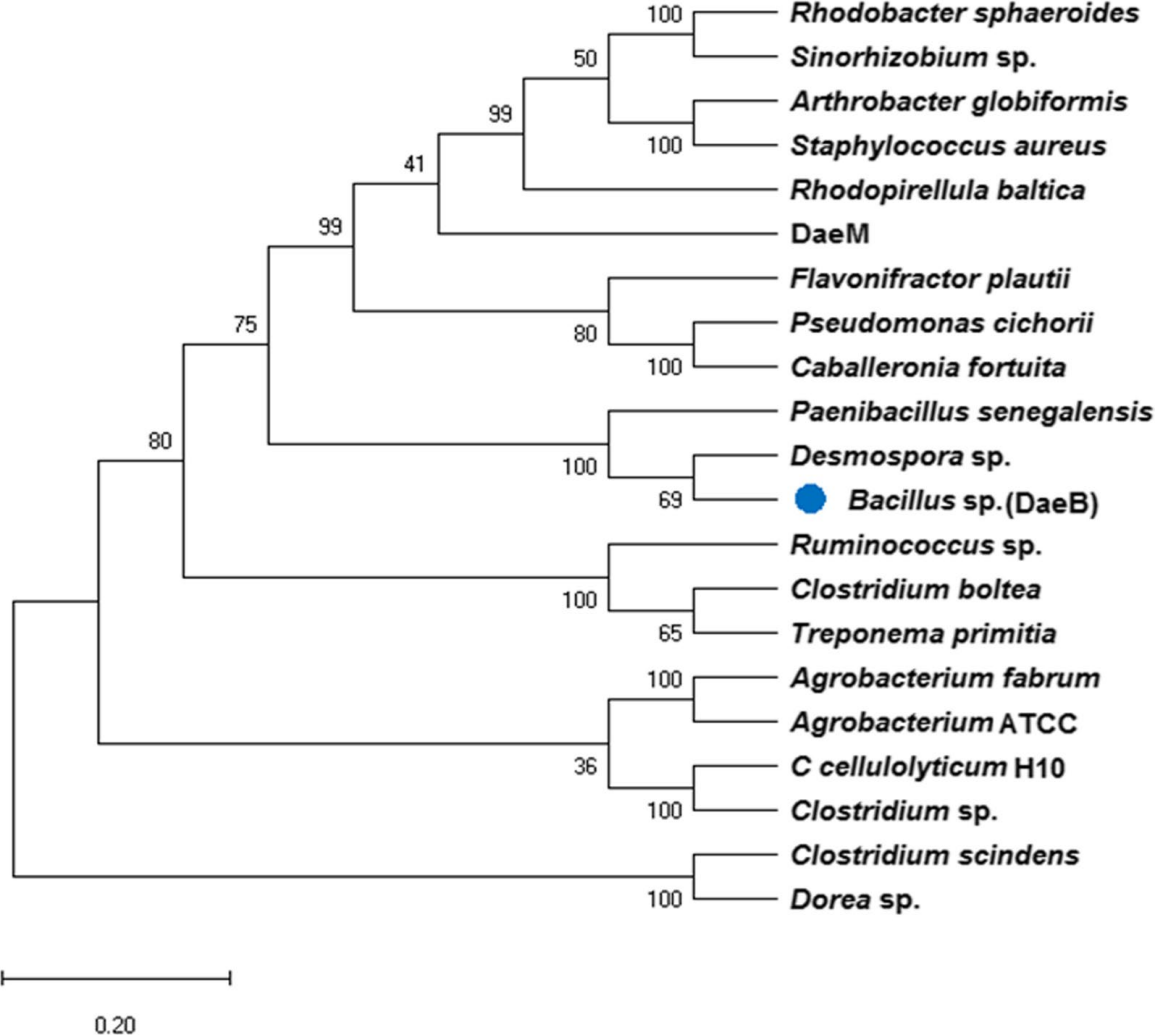
Phylogenetic relation of DaeB with the previously characterized ketose 3-epimerases. The tree was constructed using protein sequences in MEGA-X by following neighbor-joining method

**Fig. 2:**
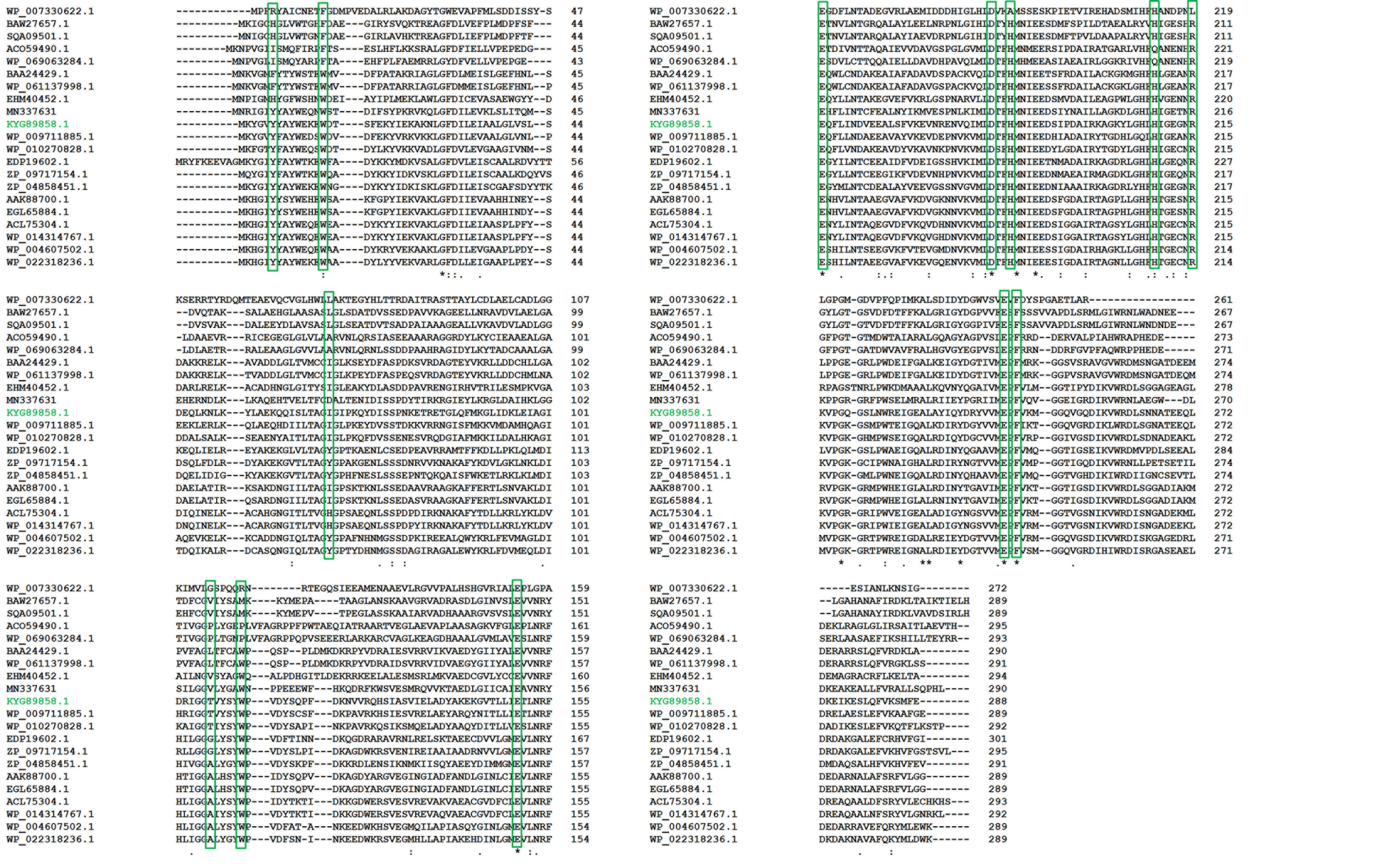
Sequence alignment among previously characterized ketose 3-epimerases. The amino acid source and GenBank accession number are as follows: *Rhodopirellula baltica* (WP_007330622.1); *Arthrobacter globiformis* (BAW27657.1); *Staphylococcus aureus* (SQA09501.1); *Rhodobacter sphaeroides* (ACO59490.1); *Sinorhizobium* sp. (WP_069063284.1); *Pseudomonas cichorii* (BAA24429.1); *Caballeronia fortuita* (WP_061137998.1); *Flavonifractor plautii (*EHM40452.1); DaeM (MN337631); *Bacillus* sp. (DaeB) (KYG89858.1); *Desmospora* sp. 8437 (WP_009711885.1); *Paenibacillus senegalensis* (WP_010270828.1); *Clostridium bolteae* (EDP19602.1); *Treponema primitia* ZAS-1 (ZP_09717154.1); *Ruminococcus* sp. (ZP_04858451.1); *Agrobacterium tumefaciens* (AAK88700.1); *Agrobacterium* sp. ATCC 31749 (EGL65884.1); *Clostridium cellulolyticum* H10 (ACL75304.1); *Clostridium scindens* (WP_004607502.1); *Dorea* sp. CAG317 (WP_022318236.1). The conserved amino acid residues critical in d-fructose epimerization are depicted in green color box

The NCBI database was mined for the identification of a novel putative d-allulose 3-epimerase (KYG89858.1) of *Bacillus* sp. origin. It showed the maximum identity of about 68 % at the protein level with a d-allulose 3-epimerase characterized from *Desmospora* sp. (Additional file 1: Fig. S1). Phylogenetic analysis of (DaeB) with previously characterized d-allulose biosynthesizing enzymes revealed an evolutionary relationship with *Desmospora* sp. and *Paenibacillus senegalensis* (Fig. 1). The conserved domain database (CDD) analysis revealed a sugar-phosphate isomerase/epimerase (55–738 amino acid residues) in DaeB (Additional file 1: Fig. S2). The multiple sequence alignment of DaeB with its homologous protein sequences divulged the conserved amino acid residues critical in d-fructose epimerization (Tyr6, Trp14, Ile66, Thr107, Trp112, Glu150, Glu156, Asp183, His186, His209, Arg215, Glu244, and Phe246) (Fig. 2).

### Secondary and tertiary structure analysis of DaeB

**Fig. 3:**
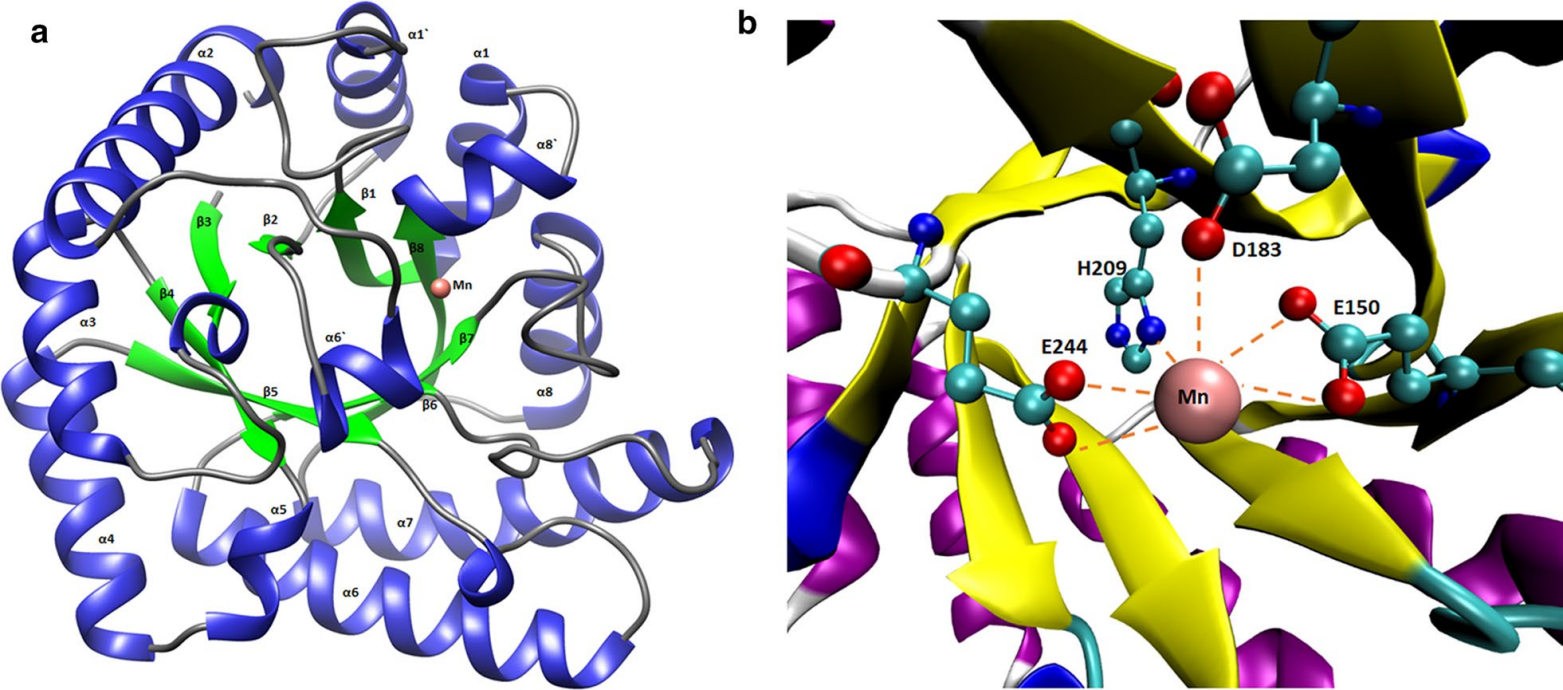
Ribbon representation of homology structure of DaeB (**a**), and stereo views of active site (**b**). The α-helix and β-strand in the Tim barrel fold are shown in blue and green color, respectively. Mn^2+^ is located in the active site with a pink sphere. The metal binding amino acid residues are shown in stick

**Fig. 4:**
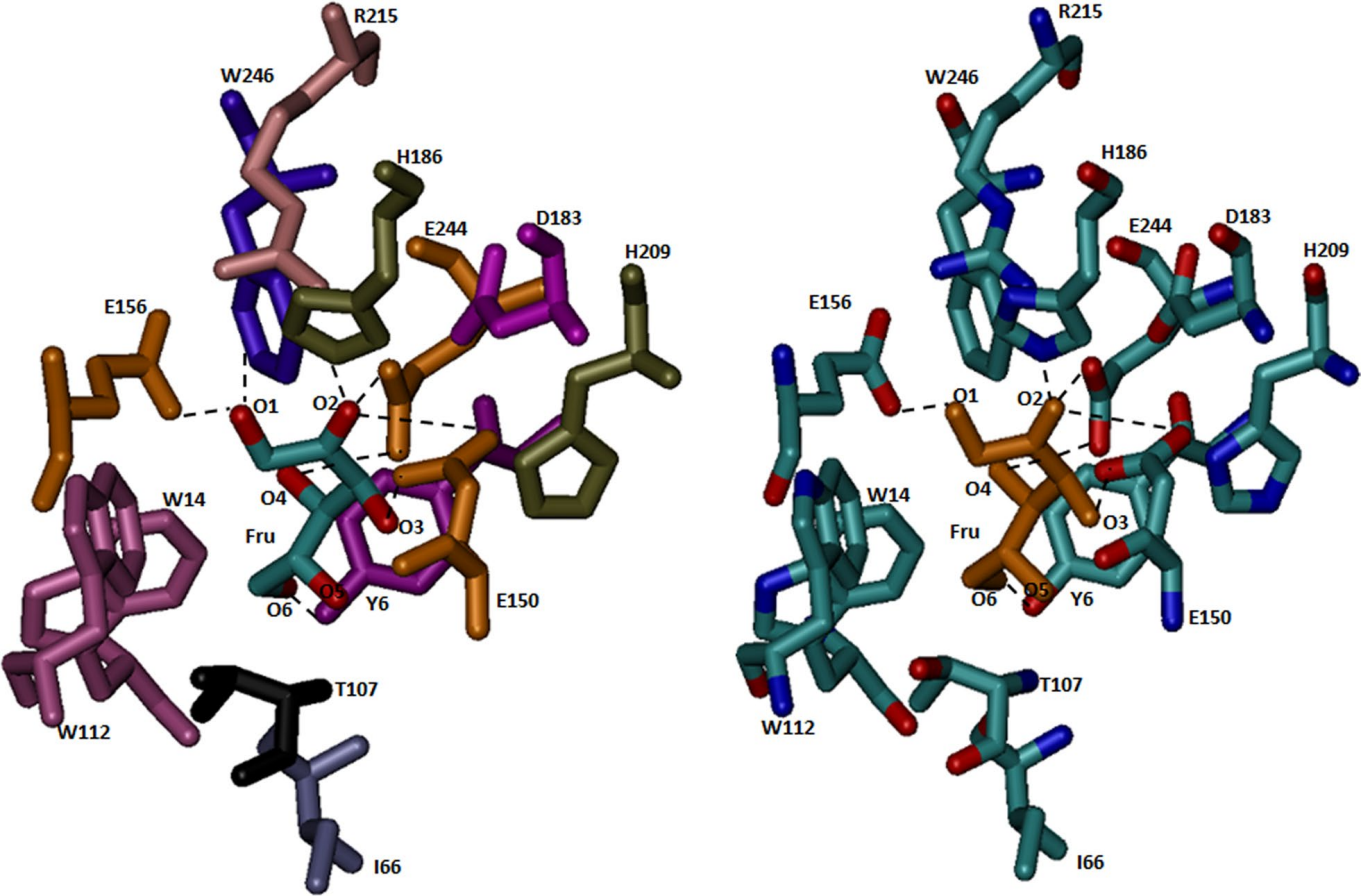
Active site residues of DaeB interacting with d-fructose molecule

Phyre2 analysis of protein sequence predicted 45 % alpha-helix, and 17 % beta-strand in DaeB (Additional file 1: Fig. S3); whereas, about 5 % sequences could not follow an appropriate structural order. A three-dimensional homology model of DaeB was generated, taking the protein structure of *Clostridium cellulolyticum* H10 d-allulose 3-epimerase (PDB No. 3VNI) as the closest template (Additional file 1: Fig. S4a). DaeB exhibits 52.78 % identity with d-allulose 3-epimerase from *C. cellulolyticum* H10 (Additional file 1: Fig. S1). A total of 288 amino acid residues of DaeB were modeled at 100 % confidence. The homology structure of DaeB could be superimposed to its template (3VNI) at the root-mean-squared-deviation of 0.000 Å (Additional file 1: Fig. S4b). The monomer structure of the DaeB is consist of eleven α-helix and eight β-strands, out of which (β/α)8 form tim barrel fold (Fig. 3a). The remaining three α-helix (α1′, α6′, α8′) are associated with the β-strand, β1, β6, and β8, respectively (Fig. 3a). At the active site, the metal (Mn) binding is coordinated by the amino acid residues, Glu150, Asp183, His209, and Glu244 (Fig. 3b). The amino acid residues possibly involved in the substrate (d-fructose) binding are depicted in Fig. 4. The O1 position (OH group) of the substrate forms hydrogen bonds with Glu156 and Arg215; while O2 position interacts with Asp183, Arg215, and Glu244, via H-bonds. The O3 position is associated with Glu150 by two hydrogen bonds, whereas, O4 and O6 interact with His209 and Tyr6 (Fig. 4). The theoretical molecular mass and pI of DaeB were computed to be 33 kDa and 4.98, respectively.

### Gene expression, protein purification, and molecular mass determination

**Fig. 5:**
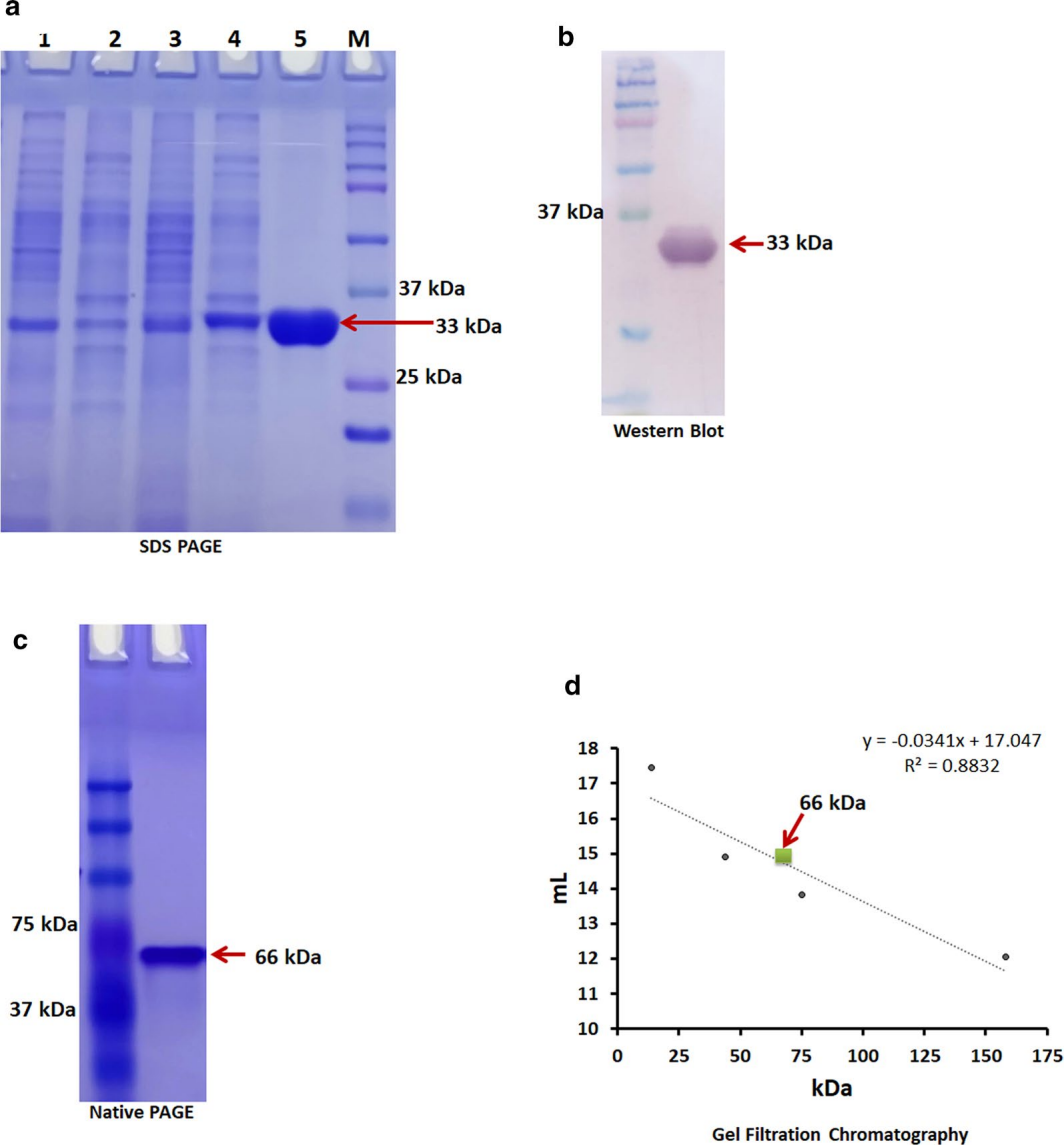
SDS-PAGE analysis of d-allulose 3-epimerase (DaeB). Lane 1: Pellet of *B. subtilis* transformed with vector; lane 2: Crude cell extract of *B. subtilis* transformed with vector; Lane 3: Pellet of *B. subtilis* expressing DaeB; Lane 4: Crude cell extract of *B. subtilis* expressing DaeB; Lane 5: Purified DaeB protein; Lane M: Protein marker (**a**). Western blot of DaeB protein using monoclonal anti-polyhistidine and antimouse IgG alkaline phosphatase antibody (**b**). Native-PAGE analysis of DaeB protein (**c**). Determination of native molecular mass of DaeB protein by gel filtration. The column was calibrated with standard proteins- Ribonuclease (13.7 kDa), Ovalbumin (44 kDa), Conalbumin (75 kDa) and Aldolase (158 kDa). DaeB is labeled with green square (**d**)

The intracellular expression of DaeB, harboring 6x-his-tag at the c-terminus, was achieved in *B. subtilis* RIK 1285 strain. SDS-PAGE analysis of purified protein estimated the molecular mass of DaeB subunits as about 33 kDa, with a purity of about 95 % (Fig. 5a). The expression of DaeB was further confirmed by western blot using the monoclonal anti-polyhistidine (primary) and anti-mouse IgG alkaline phosphatase (secondary) antibodies against Histidine region (Fig. 5b). Native PAGE analysis indicated the mass of DaeB to be about 66 kDa (Fig. 5c). In the gel filtration chromatography, DaeB protein fraction was eluted between the standard protein markers, ovalbumin (43 kDa) and conalbumin (75 kDa) (Fig. 5d), corresponding the peak to be a dimer in its native form.

### Biochemical characterization of DaeB

**Fig. 6:**
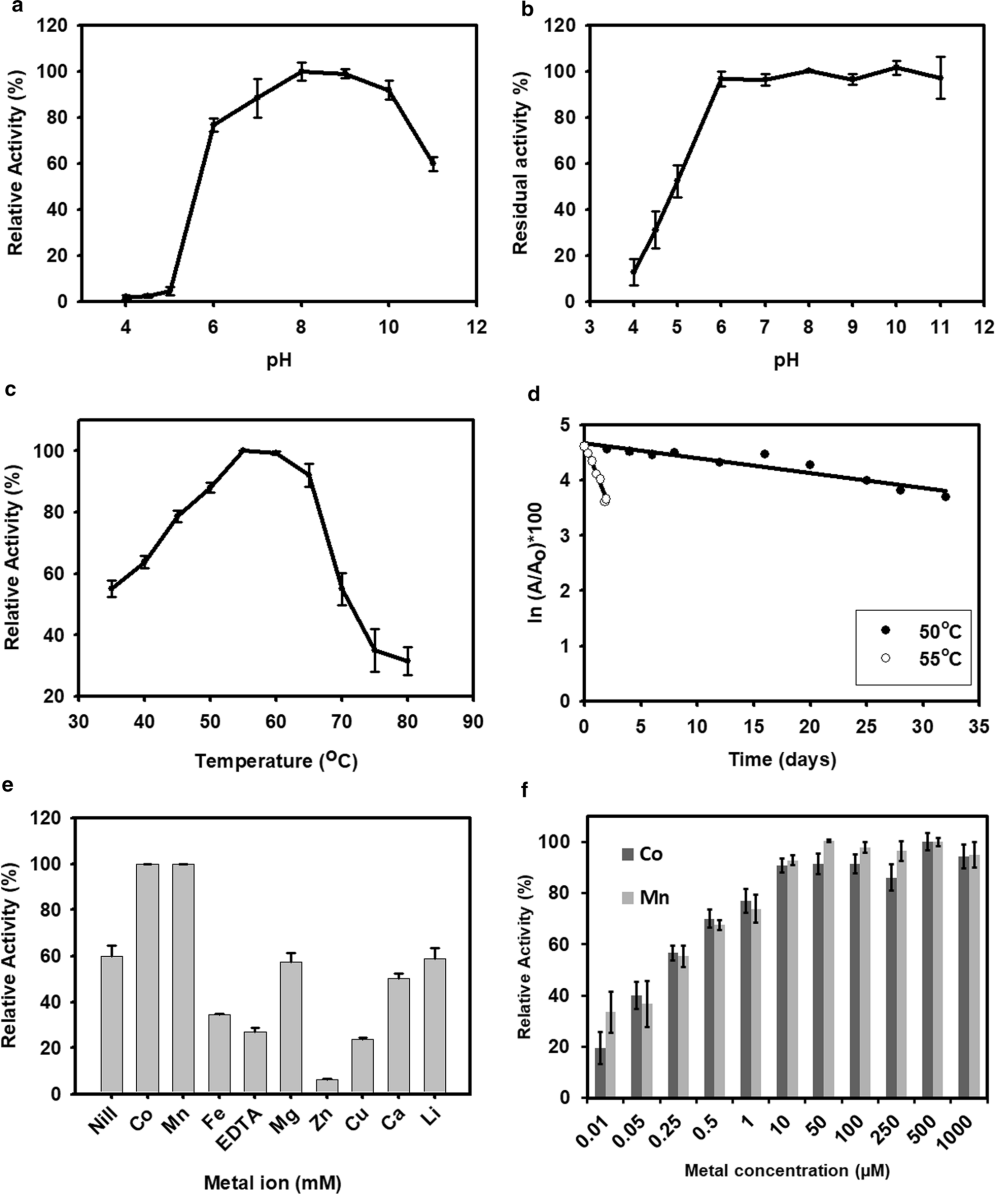
Biochemical properties of DaeB. Effect of pH (**a**), temperature (**c**), metal salts (1 mM) (**e**) and increasing concentration of MnCl_2_ and CoCl_2_ (**f**) on the activity of enzyme. Effect of pH (**b**) and temperature (50 °C and 55 °C) on the stability of enzyme (**d**). The values are mean of three biological replications ± standard deviation

The impact of pH on DaeB activity was determined over a pH range of 4.0–11.0 at 55 °C. DaeB exhibited 8.0 as the optimal pH for its activity (Fig. 6a). However, more than 75 % of its activity was displayed in the broad pH spectrum of 6.0–10.0. Moreover, DaeB was able to retain about 95 % of its initial activity after the incubation in the buffers of pH 6.0 to 11.0 for 15 h (Fig. 6b).

The temperature activity profile of DaeB depicted the maximum epimerization at 55°C, with the compromise of about 20 % reduction in the activity at 45 °C to 65 °C (Fig. 6c). However, it was able to manifest about 50 % relative activity at the temperature as low as 35  °C and as high as 70°C. DaeB protein fractions, exposed to 50 °C for an extended period of time, retained about 71 % of the initial activity after 20 days of thermal treatment. The inactivation rate constant (*k*_d_) values of DaeB were measured to be 0.027, and 0.516 at 50  °C, and 55  °C, respectively (Fig. 6d). This corresponded to the half-life (*t*_1/2_) of 25 and 1.33 days at 50  °C and 55  °C, respectively (Additional file 1: Fig. S5).

The relative activity of DaeB was measured in the presence of divalent metal ions at the final concentration of 1 mM. As shown in Fig. 6e, DaeB displayed the highest activity in the presence of Mn and Co. The presence of Mg, Ca, and Li in the reaction medium was unable to exert any remarkable effect on the enzyme’s activity. However, the presence of Zn, Cu, and Fe, and the chelating agent, EDTA, in the medium was detrimental to epimerization. Further, the availability of 50 µM Mn or 500 µM Co was noted to be sufficient in achieving the maximal catalytic activity of DaeB (Fig. 6f).

### Substrate specificity

**Fig. 7:**
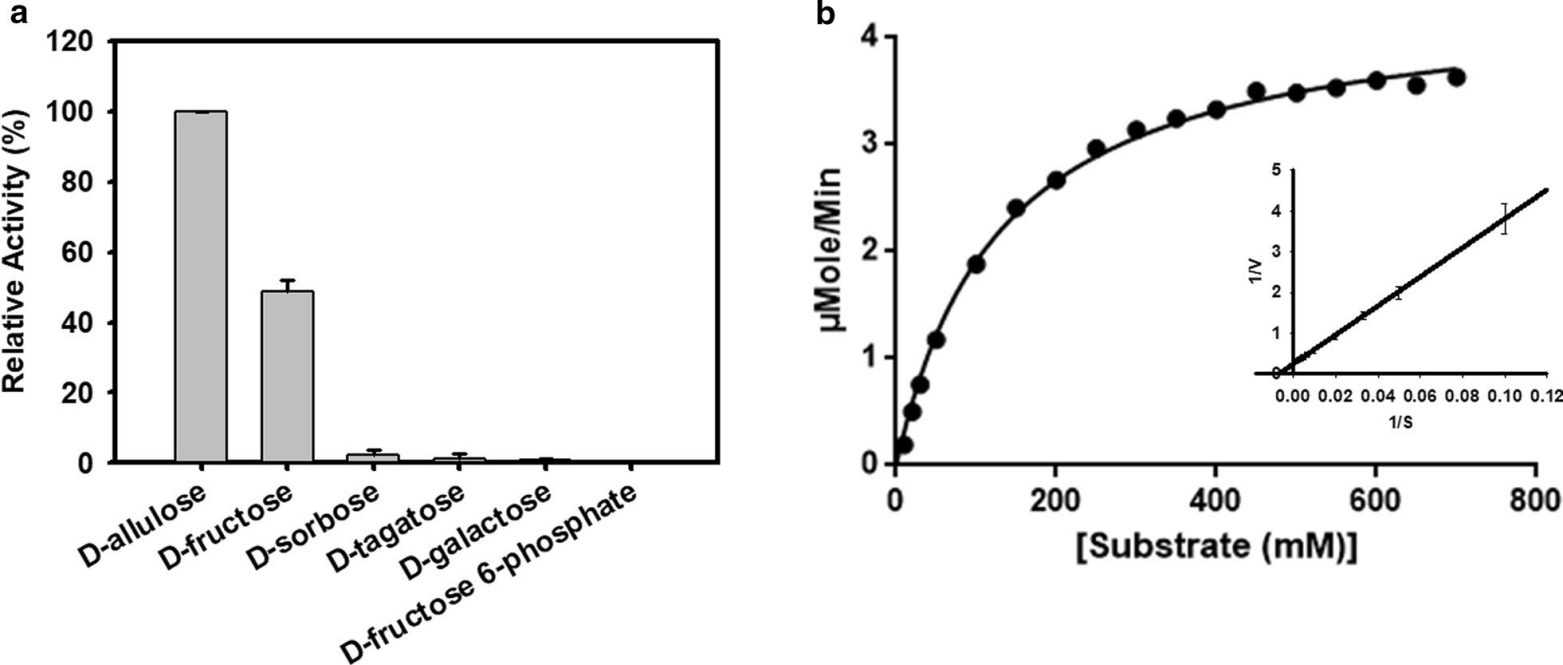
**a** Epimerization activities of DaeB towards different sugars. **b** The kinetic plot with the Michaelis-Menten equation and the Lineweaver-Burk plot. The values are mean of three biological replications ± standard deviation

The relative activity of DaeB was assessed in the presence of various substrate molecules, e.g., d-allulose, d-fructose, d-sorbose, d-tagatose, d-galactose, and d-fructose 6-phosphate. The highest relative activity of DaeB was recorded in the presence of d-allulose. The activity was 50 % higher in relation to d-fructose. Significantly reduced activity was documented in the presence of d-sorbose, d-tagatose, d-galactose, and d-fructose 6-phosphate (Fig. 7a).

### Kinetic parameters

The kinetic values of DaeB were determined towards d-fructose in terms of substrate binding efficiency, *K*_m_ = 130.6 mM, turnover number, *k*_cat_ = 367.84 s^− 1^, and catalytic efficiency, *k*_cat_/*K*_m_ = 2.81 mM^− 1^ s^− 1^ (Fig. 7b). The comparative kinetic parameters of previously characterize ketose 3-epimerases is presented in Additional file 1: Table S1.

### Storage stability

**Fig. 8:**
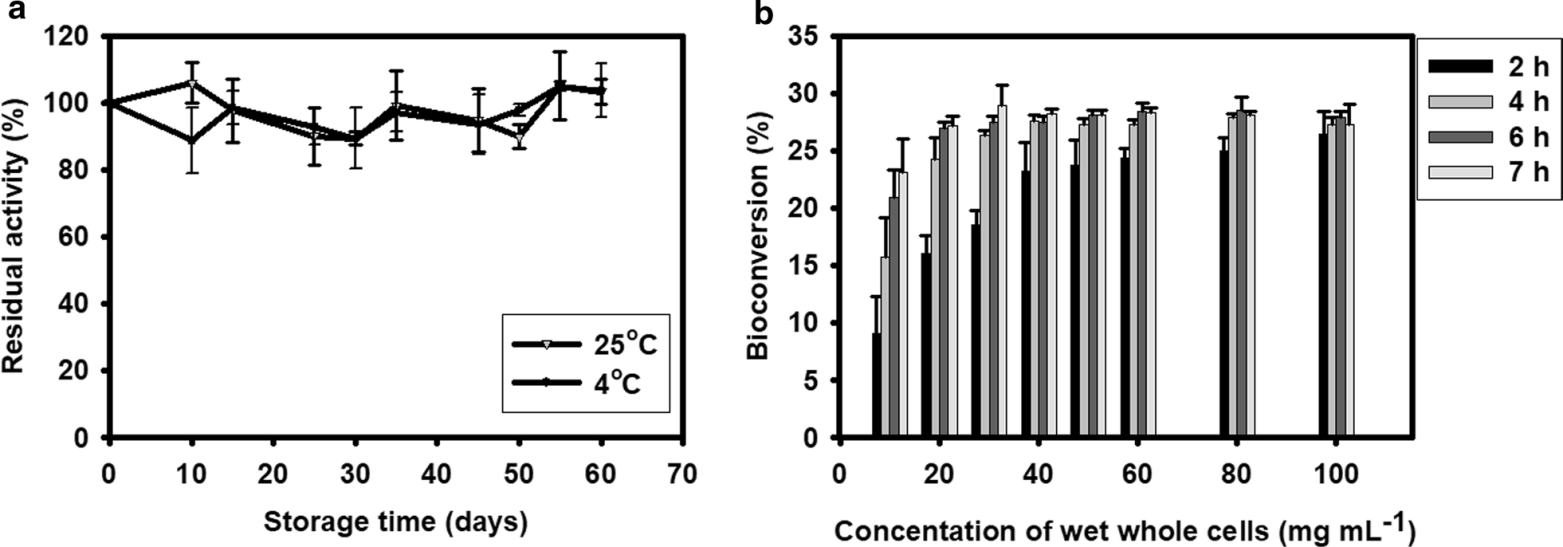
**a** Storage stability of DaeB enzyme at 4 °C and 25 °C. **b** Whole-cell bioconversion of d-allulose from 700 g L^− 1^ d-fructose. The values are mean of three biological replications ± standard deviation

The storage stability of a biocatalyst is an essential parameter from the industrial point of view. DaeB enzyme was stored in 50 mM Tris buffer at 4°C and 25°C for several months. Even after 60 days of its storage at 4 °C or 25 °C, hardly any loss in the activity was seen (Fig. 8a).

### Enzymatic biosynthesis of d-allulose

The enzymatic production of d-allulose was determined by treating 100–700 g L^− 1^ d-fructose with 0.3 g L^− 1^ DaeB at optimal reaction conditions. d-fructose to d-allulose conversion % was almost the same in the reactions performed with different substrate concentrations (Additional file 1: Fig. S6). In about 90 min, the maximum d-allulose yield of about 200 g L^− 1^ was achieved from 700 g L^− 1^, which corresponds to a conversion rate of 28.5 %. In the control reaction, executed without DaeB, no traces of spontaneous transformation of d-fructose was detected.

### Whole‐cell catalysis for d-allulose production

To avoid the process of extraction and purification of the intracellularly expressed DaeB, whole-recombinant *B. subtilis* cells were employed as a microbial cell factory for the production of d-allulose, utilizing d-fructose. The amount of recombinant cells required to execute transformation reaction was optimized by conducting whole-cell catalysis with increasing cell concentration (Fig. 8b). The solution of 700 g L^− 1^ d-fructose was incubated with 10–100 g (wet weight) L^− 1^ whole recombinant cells. The maximum d-fructose to d-allulose bioconversion of about 28 % was achieved, taking 50 g (wet weight) L^− 1^ whole-cell to treat 700 g L^− 1^ d-fructose for 6 h, yielding about 196 g of d-allulose. The control reaction, in which d-fructose solution was incubated with vector transformed *B. subtilis* cells, could not generate any trace of d-allulose.

### Bioethanol production

**Fig. 9:**
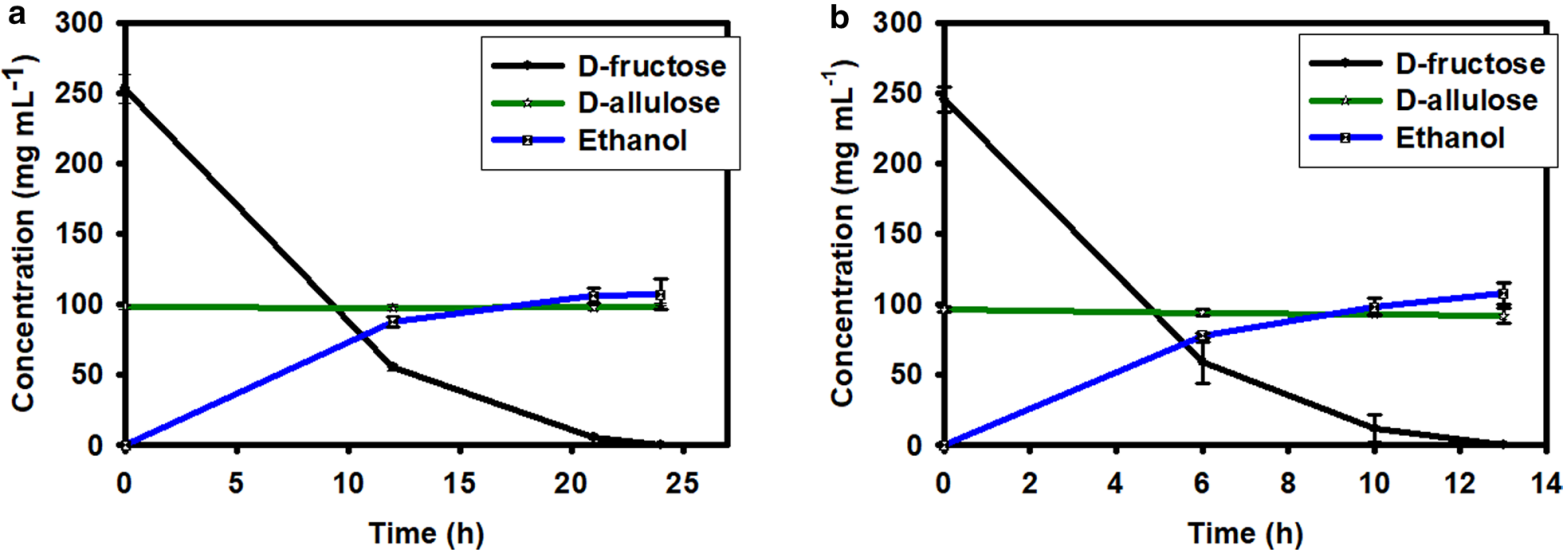
Treatment of the reaction product containing d-allulose and d-fructose using 10 % (w/v) (**a**) and 15 % (w/v) (**b**) yeast cells and formation of the by-product, bioethanol. The values are mean of three biological replications ± standard deviation

The mixture of d-fructose and d-allulose obtained from the above biocatalytic reactions was diluted by about one fold and subjected to *Saccharomyces cerevisiae* (budding yeast) led fermentation. The yeast cells, 10 % and 15 % (wet w/v), were able to consume all the fructose content in the sample in about 24 h and 13 h, respectively. Generation of about 107 g L^− 1^ ethanol was estimated from a solution containing about 250 g L^− 1^ d-fructose. This represents the ethanol yield of about approximately 42 % from d-fructose fermentation (Fig. 9a, b). Interestingly, during this fermentation reaction, the d-allulose concentration was unchanged in the solution.

## Discussion

In the present study, a novel d-allulose 3-epimerase (DaeB) was investigated from the genomic data resource from a plant probiotic strain of *Bacillus* sp. KCTC 13219. The 16 s rRNA sequence analysis of this strain signifies its phylogenetic relatedness with the genus *Lysinibacillus* [45]. The sequence alignment with DAEases from previously characterized strains established its evolutionary relationship with the genera belonging to the order Bacillales, *Desmospora* and *Paenibacillus* (Fig. 1). Nonetheless, to the best of our knowledge, this is the first report of DAEase from a strain of *Bacillus* sp.

The homology model of DaeB showed (β/α)8 TIM barrel fold and metal-binding site (Fig. 3), as found in homologous DAEases. Native mass analysis of DaeB determined it a dimer protein, similar to the family enzymes from *Pseudomonas cichorii* [46], *Rhodobacter sphaeroides* [47], *Sinorhizobium* sp. [48], and *Rhodopirellula baltica* [33]. All other DAEases had been reported to be tetramer (Additional file 1: Table S1). Sequence comparison with the known DAEases [49–52] conveyed that the amino acid residues involved in metal-binding (Glu150, Asp183, H209, and Glu244), substrate-binding (Tyr6, Trp14, Ile66, Thr107, Trp112, Glu150, Glu156, Asp183, His186, His209, Arg215, Glu244, and Phe246), and d-fructose epimerization (Glu150, and Glu244) are conserved in DaeB (Fig. 2; Additional file 1: Table S2). De-protonation and protonation of Glu150, and Glu244 execute epimerization of d-fructose to d-allulose [51]. With the greater preference towards d-allulose, DaeB has been correctly named as d-allulose 3-epimerase. Similar to *Clostridium cellulolyticum* DAEase [20], the epimerization activity was significantly low for d-tagatose and d-sorbose. A relatively greater fondness of DaeB towards d-allulose, as compared to d-fructose, could possibly be due to the formation of more hydrogen bonds between d-allulose and enzyme [51]. Furthermore, the non-allergen nature of DaeB protein sequence, as found in the *in silico* analysis (Additional file 1: Table S3), endorsed its use in the food industry.

In spite of relatively higher (61–68 %) sequence similarity of DaeB to its homologous counterparts from Bacillales, biochemical characteristics of the former were observed to be far better than previously characterized DAEases. DaeB displayed 80 % or more relative activity in the pH range of 6.0 to 10.0, unlike DAEases from *Paenibacillus senegalensis* [31], *Agrobacterium* sp. ATCC 31749 [30], and *Rhodopirellula baltica* [33] that exhibit almost nil activity at pH 6.0. Fairly good activity at slightly acidic pH, like that of *Desmospora* sp. DAEase, is a desirable property for d-fructose epimerization reaction, as it avoids browning and non-specific reaction products [53]. Similar to most of the DAEases, the presence of Co and Mn metals was observed to induce the activity of DaeB (Table 1). However, unlike the homologous proteins from *Desmospora* sp. and *Clostridium* sp., it could proffer about 60 % relative activity in the assay performed without addition of any divalent metal [23, 24]. The activity of many DAEases is remarkably diminished in the presence of Zn and Cu [17, 24, 31], whereas DaeB was noticed to tolerate 1 mM Cu by exhibiting about 25 % relative activity. This could be ascribed to the stable structure of the protein. Tolerance to metals is an expedient trait for the industrial application of the biocatalyst. The metal tolerance feature of DaeB may be of its potential use in the enzymatic treatment of the feedstock containing metals such as Ca, Mg, Fe, etc.

**Table 1:** Comparisons of biochemical properties of DaeB and previously characterized d-allulose biosynthesizing enzymes

Enzyme	Enzyme sources	GenBank accession number	Optimum pH	Optimum temperature (°C)	Cofactor	Most suitable substrate	Half- life (minutes)	Turnover number *k*_cat_ (s^− 1^)	Equilibrium (d-allulose to d-fructose)	References
d-tagatose 3-epimerase (DTEase)	*Pseudomonas cichorii*	BAA24429.1	7.5	60	None	d-tagatose	NR	NR	20:80 (30 °C)	[41]
	*Caballeronia fortuita*	WP_061137998.1	7.5	65	Co^2+^	d-tagatose	63 (60 °C) 307 (55 °C)	157.21	28.1:71.9	[12]
	*Sinorhizobium* sp.	WP_069063284.1	8.0	50	Mn^2+^	d-tagatose	NR	50	25.3:74.7	[43]
d-allulose 3-epimerase (DAEase)	*Agrobacterium tumefaciens*	AAK88700.1	8.0	50	Mn^2+^	d-allulose	8.9 (55 °C), 64 (50 °C)	34.46	32: 68 (30 °C)	[14]
	*Clostridium cellulolyticum* H10	ACL75304.1	8.0	55	Co^2+^	d-allulose	408 (60 °C)	55.9	32: 68 (55 °C)	[15]
	*Ruminococcus* sp.	ZP_04858451.1	7.5–8.0	60	Mn^2+^	d-allulose	~96 (60 °C)	59.4	28:72 (60 °C)	[16]
	*Clostridium scindens*	WP_004607502.1	7.5	60	Mn^2+^	d-allulose	50 (60 °C), 108 (50 °C)	5.8	28: 72 (50 °C)	[17]
	*Clostridium* sp.	WP_014314767.1	8.0	65	Co^2+^	d-allulose	15 (60 °C)	272	28: 72 (65 °C)	[18]
	*Desmospora* sp. 8437	WP_009711885.1	7.5	60	Co^2+^	d-allulose	120 (50 °C)	1059.55	30:70 (60 °C)	[19]
	*Clostridium bolteae*	EDP19602.1	7.0	55	Co^2+^	d-allulose	156 (55 °C)	59	31: 69 (55 °C) 32: 68 (60 °C)	[20]
	*Dorea* sp. CAG317	WP_022318236.1	6.0	70	Co^2+^	d-allulose	30 (60 °C)	NR	30:70 (70 °C)	[21]
	*Treponema primitia* ZAS-1	ZP_09717154.1	8.0	70	Co^2+^	d-allulose	30 (50 °C)	292	28: 72 (70 °C)	[22]
	*Flavonifractor plautii*	EHM40452.1	7.0	65	Co^2+^	d-allulose	40 (65 °C)	342	31:69	[23]
	*Arthrobacter globiformis*	BAW27657.1	7.0–8.0	70	Mg^2+^	d-allulose	Not reported	41.8	24: 76 (30 °C) 27: 73 (70 °C)	[24]
	*Agrobacterium* sp. ATCC 31749	EGL65884.1	7.5–8.0	55–60	Co^2+^	d-allulose	267 (55 °C), 28.2 (60 °C), 3.8 (65 °C)	106.7	30:70 (55 °C)	[25]
	*Paenibacillus senegalensis*	WP_010270828.1	8.0	55	Co^2+^, Mn^2+^	d-allulose	140 (60 °C)	40.92	30:70	[26]
	*Staphylococcus aureus*	SQA09501.1	7.5	70	Mg^2+^	d-allulose	120 (70 °C)	22.9	38.9:61.1	[27]
	*Rhodopirellula baltica*	WP_007330622.1	8.0	60	Mn^2+^	d-allulose	52 (60 °C)	14.90	28.6:61.4	[28]
	DaeM	MN337631.	7.0	80	Co^2+^	d-allulose	9900 (60 °C) 3240 (70 °C) 49 (80 °C)	41.99	31:69	[32]
	*Bacillus* sp. (DaeB)	KYG89858.1	8.0	55	Mn ^2+^	d-allulose	36,000 (50 °C) 1320 (55 °C)	367	28.5:71.5	This study
d-fructose 3-epimerase (DFEase)	*Rhodobacter sphaeroides*	ACO59490.1	9.0	40	Mn^2+^	d-fructose	60 (50 °C)	NR	23:77 (40 °C)	[42]
L-ribulose 3-epimerase (LREase)	*Mesorhizobium loti*	BAN15042.1	8.0	60	Mn^2+^	L-ribulose	Not reported	NR	NR	[13]

The temperature activity profile of DaeB was similar to many DAEases, disposing higher level (80 %) of relative activity in the range of 50–65 °C (Table 1). Thermal activity at higher temperatures of this enzyme is advantageous in increasing the solubility of substrate and product, and avoiding the contamination possibilities in the reaction [37]. DaeB is able to withstand thermal treatment, exhibiting a half-life of about 25 days at 50 °C. Such a high level of thermal stability at 50 °C has not been reported in case of any DAEases characterized from diverse cultivable bacterial genera (Table 1). This is a worthwhile trait of a biocatalyst that propounds the economic viability of the enzymatic process at pilot scale. The higher magnitude of thermal stability in DaeB could be ascribed to a rigid configuration of the amino acid residues and multiple hydrophobic interactions in the protein [11, 51]. Further, the occurrence of fairly high number of acidic amino acid residues on the surface of DaeB (Additional file 1: Fig. S7, Table S4) could contribute to a better protein solubility and high storage stability of the enzyme [54]. Besides reasonably higher thermal stability and storage stability, the elevated turnover number is another crucial trait for an enzyme. The *k*_cat_ of DaeB was better than several previously reported DAEases, except that of *Desmospora* sp. 8437 (Table 1; Additional file 1: Table S1). Notwithstanding, a DAEase with this extent of heat stability and catalytic efficiency is not reported so far. These traits make DaeB a prudent biocatalyst for industrial use.

In this study, *B. subtilis* cells expressing DaeB were demonstrated to be capable of executing whole-cell transformation reaction, achieving d-fructose to d-allulose conversion. This process offers D-allulose biosynthesis devoid of tedious extraction and purification of the enzyme [31, 55]. The d-allulose yield of about 28 % by whole-cell transformation method was comparable with the previous reports [31, 37]. Strategies have been developed for the accomplishment of conversion shift for a greater d-allulose yield. The use of borate had been exemplified to make d-allulose-borate complex in the DAEase reaction, leading to the conversion of approximately 60 % d-fructose into d-allulose [56]. In a recent study, d-allulose had been subjected to transformation into d-allulose 1-phosphate by the catalytic action of L-rhamnulose kinase, achieving the conversion of up to 99 % d-fructose [57]. Another option could be the transformation of the residual d-fructose into ethanol [58, 59]. In accordance with the previous studies, in the present investigation, after DaeB reaction, the remaining proportion of d-fructose (72 %) in the catalytic product was converted into ethanol by the interference of yeast fermentation. The co-production of d-allulose and ethanol from the low-cost biomass could be techno-economically feasible [52, 58, 60]. However, the reaction product containing the mixture of d-fructose and d-allulose may be subjected to chromatography separation of the two epimers by employing Simulated Moving Bed Chromatography (SMBC), obtaining d-allulose of about 99 % purity [61, 62]. The fraction of d-fructose thus obtained may further be recycled for d-allulose production.

## Conclusions

This study identified and characterized a novel d-allulose 3-epimerase (DaeB) from a plant probiotic strain, *Bacillus* sp. KCTC 13219. The protein showed close phylogenetic association with the DAEases reported from other genera of Bacillales, *Desmospora* and *Paenibacillus*. However, DaeB proclaimed superior enzymatic traits as compared to the DAEases characterized so far. It has excessive thermostability along with an elevated turnover number. Furthermore, the *B. subtilis* cells expressed with this biocatalyst (DaeB) was found suitable for executing whole-cell biotransformation reaction. The results manifested that DaeB has enormous potential for pilot-scale manufacturing of the rare functional sugar, d-allulose.

## Methods

### Gene identification and sequence analysis

An uncharacterized protein from *Bacillus* sp. KCTC 13219, KYG89858.1, predicted as dolichol monophosphate mannose synthase in the NCBI database, was selected (in this study named as DaeB). The conserved domain analysis was done by using NCBI conserved domain database (CDD). The protein sequence was evaluated for identification of conserved residues by doing sequence comparison (Clustal Omega multiple sequence alignment) with previously characterized d-allulose 3-epimerases. The phylogenetic tree was constructed by MEGA X taking the protein sequences of homologous d-allulose 3-epimerases as input. The Neighbor-Joining method was used with the bootstrap replications of 1000 and the evolutionary distances were calculated using the Poisson-Correction method [63]. Allergenicity prediction was assessed by implementing allergenic predication tool such as Sortaller [64], AllerTOP [65], Allermatch [66], and SDAP [67].

### Structure modeling of DaeB

A three-dimensional (3D) homology model of DaeB was constructed using Modeller 9.17 software. The crystal structure of *Clostridium cellulolyticum* H10 (PDB ID: 3VNI, 1.98 Å) was selected as a template. The align2d auto model commands were used to generate homology model. The homology structure was refined via annealing MD simulation. Based on the value of DOPE assessment score and modeller objective function, the best model was selected. The model structure was visualized using Visual Molecular Dynamics version 1.9.3. The protein structure was analyzed by using Phyre2 protein fold recognition server [68]. Superimposition of homology model over its template was done using the UCSF Chimera software (https://www.cgl.ucsf.edu/chimera). The surface accessibility of acidic amino acid residues was predicted by NetsurfP tool.

### Cloning of *Bacillus* sp. d-allulose 3-epimerase (*daeB*)

The putative d-allulose 3-epimerase (*daeB*) gene was codon optimized for *Bacillus subtilis*, and chemically synthesized. The gene was cloned into the pBES vector (Takarabio, Kusatsu, Shiga, Japan) between the restriction sites, *Mlu*I and *Xba*I. The gene construct was introduced in *B. subtilis* RIK 1285 for intracellular expression.

### Gene expression and purification of DaeB

The *B. subtilis* cells transformed with the construct (pBES-*daeB*) were cultured in Luria broth (LB) medium, containing 10 µg mL^− 1^ kanamycin, at 37°C, 100 RPM for 24 h, for gene expression. The cells were harvested by centrifugation at 6,000 RPM for 8 min at room temperature (RT). The cell pellet was suspended in 50 mM Tris buffer of pH 8, containing 300 mM NaCl, and 10 mM Imidazole, followed by disruption of the cells by sonication for 3 min (3 second on and 10 second off with the cut of 25°C temperature). The cell debris was removed by centrifugation at 10,000 RPM for 30 min at 4°C. The cells lysate was passed through the 0.45 µM filter (Axiva, New Delhi, India). The recombinant protein was purified using the AKTA pure system (GE Healthcare, Chicago, Illinois, United States). The crude lysate filtrate was passed through a pre-equilibrated HisTrap FF column (GE Healthcare), preloaded with nickel. The composition of equilibrium buffer was 50 mM Tris buffer of pH 8.0, containing 300 mM NaCl, and 10 mM Imidazole. The unbound proteins were washed with the wash buffer (50 mM Tris buffer of pH 8.0, 300 mM NaCl, and 40 mM Imidazole), and the 6 × histidine-tagged recombinant protein was eluted using the elution buffer (50 mM Tris buffer of pH 8.0, 300 mM NaCl, and 300 mM Imidazole). The quantitative measurement of the protein was done by the Bradford assay, using the bovine serum albumin as standard.

### Molecular mass determination

The native molecular mass of the DaeB was determined *via* gel filtration chromatography and native PAGE analysis. The Superdex 200 Increase 10/300 GL column (bed volume 24 mL, GE Healthcare) was equilibrated with 50 mM Tris buffer (8.0 pH), and 300 mM NaCl. The purified DaeB was passed through the column at the flow rate of 0.5 mL min^− 1^. In order to estimate the native mass of the protein, standard protein markers including ribonuclease A (13.7 kDa), ovalbumin (44 kDa), conalbumin (75 kDa), and aldolase (158 kDa), were analyzed using the same column. Native Polyacrylamide Gel Electrophoresis (PAGE) of 5 % stacking gel and 10 % resolving gel was also used to estimate the native mass of DaeB. The purity and subunit molecular mass of DaeB was assessed by Sodium Dodecyl Sulphate (SDS) PAGE using a 5 % stacking gel and 12 % resolving gel. The staining of protein gel was performed using the brilliant blue-R dye. The de-staining of gel was done by using an aqueous solution of 10 % (v/v) glacial acetic acid, and 7.5 % (v/v) methanol.

### Western blotting

The purified protein was run in SDS-PAGE (12 %), followed by blotting onto a nitrocellulose membrane (Bio-Rad, Hercules, CA, USA). The membrane was blocked with 3 % BSA in 1X TBST buffer (pH 7.6). The membrane was washed three times with 1X TBST buffer. Then the membrane was incubated with monoclonal anti-polyhistidine antibody (Sigma, St. Louis, MO, USA) at 4 °C for 12 h, followed by thorough washing with 1X TBST buffer. After this, anti-mouse IgG alkaline phosphatase (secondary antibody) (Sigma, St. Louis, MO, USA) was introduced, and incubated at room temperature for 2 h. After washing with 1X TBST buffer, the membrane protein was visualized by 5-bromo-4-chloro-3-indolylphosphate/nitroblue tetrazolium) substrate (Sigma, St. Louis, MO, USA) at room temperature [37, 69].

### Enzyme activity assay

The enzyme activity was calculated as a measure of d-allulose produced from d-fructose. The enzymatic assay was performed in 50 mM Tris buffer (pH 8.0) containing 100 mM d-fructose as substrate, 50 µM Mn, and 0.025 mg mL^− 1^ DaeB enzyme, at 55  °C for 5 min. The reaction was ceased by heating at 100  °C for 10 min. The amount of d-allulose was determined by high performance liquid chromatography (HPLC). A unit of enzyme was determined as the amount of enzyme required to catalyze the formation 1 µmol d-allulose min^− 1^ under optimal reaction conditions. All the experiments were performed in triplicate and the average data were represented with standard deviation.

### Effect of metal ions

To determine the effect of metal ions on the activity of DaeB, reaction assays were performed in the presence of 1 mM CoCl_2_, MnCl_2_, FeCl_2_, MgCl_2_, ZnCl_2_, CuCl_2_, CaCl_2_, LiCl_2_, and EDTA at standard reaction condition. Reaction performed without addition of metal ions was defined as control. Effect of increasing concentration of MnCl_2_ and CoCl_2_ (10 nM to 1000 µM) was also examined.

### Effect of pH and temperature

To investigate the effect of the pH on the activity of DaeB, reaction assays were conducted in the buffers of different pH range, e.g., 50 mM sodium acetate (pH 4.0, 4.5, and 5.0), 50 mM HEPES (pH 6.0), 50 mM Tris (pH 7.0, 8.0, and 9.0), and 50 mM Glycine NaOH (pH 10.0, and 11.0), at 55 °C for 5 min. The effect of temperature on the activity of DaeB was studied by performing enzyme assays in 50 mM Tris buffer of pH 8.0 (containing 50 µM Mn) at a wide range of temperature, 35–80 °C, for 5 min.

The pH stability of DaeB was examined by incubating the protein in the aforementioned buffers of different pH at 4°C for 15 h. Standard enzyme assays were performed with the pre-exposed enzyme fractions. Heat stability was measured by exposing DaeB in 50 mM Tris buffer of pH 8.0 (containing Mn) to 50°C and 55°C for a longer period of time, and enzyme assays were performed at different time intervals. To determine the inactivation rate constant (*k*_d_), the residual activity (%) of DaeB were plotted against time in a semi-log manner. The half-life of the enzyme was calculated by analyzing the first-order inactivation kinetic model. The values for *t*_1/2_ were calculated from the equation, *t*_1/2_ = ln(2)/*k*_d_, where *k*_*d*_ denotes inactivation rate constant, and ln(2) represents the natural logarithm of 2.

### HPLC analysis

The quantitation of the product, d-allulose, was performed by High performance liquid chromatography (HPLC) system (Waters Acquity, Milford, Massachusetts), equipped with refractive index detector and Hi-Plex-Ca column (column temperature 85°C). Deionized water was used as mobile phase. The injection volume was 20 µL. The flow rate was set as 0.6 mL min^− 1^.

### Kinetic parameters

The kinetic parameters of DaeB were computed by performing standard enzyme assays in the presence of increasing concentrations of the substrate (d-fructose) ranging from 10 to 700 mM. The kinetic parameters including Michaelis-Menten constant (*K*_m_), turnover number (*k*_cat_), and catalytic efficiency (*k*_cat_/*K*_m_) were calculated using the Lineweaver-Burk plots of the Michaelis-Menten equation.

### Substrate specificity and storage stability

The substrate specificity of DaeB was examined by conducting standard reaction assays, taking (100 mM) different sugar substrates, e.g., d-allulose, d-fructose, d-tagatose, d-galactose, d-sorbose, and d-fructose 6-phosphate.

The storage stability of DaeB was inspected by keeping the enzyme in 50 mM Tris buffer containing the 50 µM MnCl_2_ at 4°C and 25°C for several months. The residual activity of stored enzyme was measured at different time points.

### Whole‐cell reaction

The recombinant *B. subtilis* cells transformed with *daeB* construct were cultivated as described above. The cells were harvested by centrifugation at 6,000 RPM for 8 min at room temperature. The cell pellet was re-suspended and washed with 10 mM Tris buffer pH 8.0. The solution of 700 mg mL^− 1^ d-fructose in 10 mM Tris buffer (pH 8.0), containing 50 µM of MnCl_2_, was treated with 10–100 mg mL^− 1^ whole-cell fractions at 55°C and 100 RPM for 2–7 h. The reaction volume was 100 mL. After treatment, the cells were removed from the reaction product by centrifugation (10,000 RPM for 5 min) and microfiltration, followed by boiling at 100°C for 10 min. *B. subtilis* cells transformed with blank vector (pBES) were taken as control.

### Ethanol production

The baker’s yeast (*Saccharomyces cerevisiae* MTCC No. 36) was cultured in the malt extract broth (malt extract 3 g L^− 1^, yeast extract 3 g L^− 1^, peptone 5 g L^− 1^, and glucose 10 g L^− 1^) (pH 6.2) at 28 °C and 150 RPM for 24–30 h. The yeast cells were harvested by centrifuge at 6000 RPM for 5 min at room temperature. The reaction sample containing the mixture of d-allulose and d-fructose produced was treated with the baker’s yeast for the fermentation of remaining fructose. The yeast cells (10 % and 15 % wet w/v) were added into the reaction product, i.e., mixture of d-fructose and d-allulose, and incubated at 28°C, 100 RPM for 25 h for the fermentation of d-fructose and production of ethanol as a by-product.

## Supplementary Information

**Additional file 1:** Figure S1. Identity (%) matrix of DaeB with the previously characterized ketose 3-epimerases. Figure S2. Conserved domain analysis in DaeB protein. Figure S3. Predicted secondary structure of DaeB protein sequence. The information has been collected by Phyre2 web-based service. Figure S4. (A) A predicted 3D homology model of DaeB. A total of 288 amino acid residues of DaeB were modelled at 100% confidence. (B) Superimposition of the homology model of DaeB over the tertiary structure of *C. cellulolyticum* H10 (RMSD value 0.0Å). Figure S5. Effect of temperature on the heat stability of DaeB at pH 8.0. Figure S6. Time-point conversion of D-allulose from D-fructose (100-700 g L^−1^) using DaeB enzyme. Figure S7. Homology model of DaeB showing acidic amino acid residues (Asp and Glu) on the surface of the protein. Table S1. Comparison of native molecular mass, kinetic parameters, and specific activity of DaeB and other ketose 3-epimerases. Table S2. The conserved active site amino acid residues in the ketose 3-epimerases for which protein structure has been determined, and the homologous residues identified in DaeB. Table S3. In silco determination of allergenicity in DaeB protein. Table S4. Total number of acidic amino acid residues (Asp+Glu), and acidic amino acid residues on the protein's surface of DAEases.

## Data Availability

Not applicable.

## Abbreviations

DaeB: *Bacillus* sp. d-allulose 3-epimerase
°C: Degree celsius
*k*_cat_: Turnover number
g L^−^^1^: Gram per litre
FDA: Food and Drug Administration
GRAS: Generally regarded as safe: DAEase: d-allulose 3-epimerases
pI: Isoelectric point
kDa: Kilo dalton
SDS: Sodium dodecyl sulfate
PAGE: Polyacrylamide gel electrophoresis
*k*_d_: Inactivation rate constant
*t*_1/2_: Half-life
µM: Micromoler
*K*_m_: Michaelis mention constant
*k*_cat_/*K*_m_: Catalytic efficiency
mM: Milimolar
CDD: Conserved domain database
LB: Luria broth
RT: Room temperature
