# Supplementary material for: d-Allulose 3-epimerase of Bacillus sp. origin manifests profuse heat‐stability and noteworthy potential of d-fructose epimerization
Source: Microb Cell Fact. 2021 Mar 4;20:60. doi: 10.1186/s12934-021-01550-1 (PMC7934257; doi:10.1186/s12934-021-01550-1)
Supplement: Supplementary file 1 — Additional file 1: Figure S1. Identity (%) matrix of DaeB with the previously characterized ketose 3-epimerases. Figure S2. Conserved domain analysis in DaeB protein. Figure S3. Predicted secondary structure of DaeB protein sequence. The information has been collected by Phyre2 web-based service. Figure S4. (A) A predicted 3D homology model of DaeB. A total of 288 amino acid residues of DaeB were modelled at 100% confidence. (B) Superimposition of the homology model of DaeB over the tertiary structure of C. cellulolyticum H10 (RMSD value 0.0Å). Figure S5. Effect of temperature on the heat stability of DaeB at pH 8.0. Figure S6. Time-point conversion of D-allulose from D-fructose (100-700 g L−1) using DaeB enzyme. Figure S7. Homology model of DaeB showing acidic amino acid residues (Asp and Glu) on the surface of the protein. Table S1. Comparison of native molecular mass, kinetic parameters, and specific activity of DaeB and other ketose 3-epimerases. Table S2. The conserved active site amino acid residues in the ketose 3-epimerases for which protein structure has been determined, and the homologous residues identified in DaeB. Table S3. In silco determination of allergenicity in DaeB protein. Table S4. Total number of acidic amino acid residues (Asp+Glu), and acidic amino acid residues on the protein's surface of DAEases. [file 12934_2021_1550_MOESM1_ESM.pdf]

D-allulose 3-epimerase of *Bacillus* sp. origin manifests profuse heat-stability and noteworthy potential of D-fructose epimerization

Satya Narayan Patel[1], Girija Kaushal[1], Sudhir P. Singh[1,*]

[1]Center of Innovative and Applied Bioprocessing, S.A.S. Nagar, Sector-81 (Knowledge City) Mohali-140306, India

*Corresponding author
*Sudhir P. Singh: sudhirsingh@ciab.res.in

Gene Source	*R. baltica*	*A. globiformis*	*S. aureus*	*R. sphaeroides*	*Sinorhizobium* sp.	*P. cichorii*	*C. fortuita*	*F. plautii*	*daeM*	*Bacillus* sp. (DaeB)*	*Desmospora* sp.	*P. senegalensis*	*C. boltea*	*T. primitia*	*Ruminococcus* sp.	*A. fabrum*	*Agrobacterium* ATCC	*C. cellulolyticum*	*Clostridium* sp.	*C. scindens*	*Dorea* sp.
R. baltica	100	19.77	20.93	22.69	20.46	22.14	20.23	18.63	23.17	18.15	19.69	19.69	21.54	21.92	19.62	21.62	21.24	22.01	22.39	21.71	23.26
A. globiformis	19.77	100	78.89	27.66	28.47	31.52	30.32	27.24	31.07	28.06	30.11	28.72	26.52	27.92	26.88	30.47	30.11	26.86	27.3	28.32	27.96
S. aureus	20.93	78.89	100	26.6	27.76	28.99	27.8	26.16	30	25.9	27.6	27.3	24.37	26.5	23.66	28.32	27.96	26.5	26.6	25.45	26.16
R. sphaeroides	22.69	27.66	26.6	100	59.73	30.04	29.58	27.72	28.32	27.4	27.3	28.77	29.93	29.02	27.66	31.91	31.91	29.02	29.12	30.5	30.5
***Sinorhizobium* sp.**	20.46	28.47	27.76	59.73	100	27.66	26.5	26.41	25.96	25.71	25.62	26.06	27.3	28.07	24.91	29.89	29.18	26.32	25.35	26.33	25.98
P. cichorii	22.14	31.52	28.99	30.04	27.66	100	93.1	44.83	41.9	39.51	42.31	40.21	36.24	35.31	34.27	40.21	40.56	42.66	42.31	39.65	38.25
C. fortuita	20.23	30.32	27.8	29.58	26.5	93.1	100	43.1	40	39.51	41.81	39.72	35.42	34.49	34.84	39.72	40.07	40.42	41.11	39.51	37.06
F. plautii	18.63	27.24	26.16	27.72	26.41	44.83	43.1	100	39.86	36.81	37.85	39.24	37.72	38.19	36.81	44.79	44.44	44.79	44.79	42.51	39.37
daeM	23.17	31.07	30	28.32	25.96	41.9	40	39.86	100	42.81	44.06	43.6	39.02	41.87	40.56	41.61	41.61	44.98	44.29	41.96	41.61
Bacillus* sp. (DaeB)	18.15	28.06	25.9	27.4	25.71	39.51	39.51	36.81	42.81	100	68.06	61.46	47.57	50	49.31	47.57	47.57	52.78	53.12	50.87	51.22
***Desmospora* sp.**	19.69	30.11	27.6	27.3	25.62	42.31	41.81	37.85	44.06	68.06	100	68.17	47.4	52.25	48.79	49.83	50.17	49.83	50.87	50.35	50.35
P. senegalensis	19.69	28.72	27.3	28.77	26.06	40.21	39.72	39.24	43.6	61.46	68.17	100	46.37	52.4	48.79	51.56	51.56	50.34	51.71	51.56	52.25
C. boltea	21.54	26.52	24.37	29.93	27.3	36.24	35.42	37.72	39.02	47.57	47.4	46.37	100	68.04	62.54	49.48	49.13	52.94	53.98	51.74	52.43
T. primitia	21.92	27.92	26.5	29.02	28.07	35.31	34.49	38.19	41.87	50	52.25	52.4	68.04	100	62.54	55.36	55.02	58.36	59.25	55.02	56.06
***Ruminococcus* sp.**	19.62	26.88	23.66	27.66	24.91	34.27	34.84	36.81	40.56	49.31	48.79	48.79	62.54	62.54	100	50.52	49.83	52.25	52.94	52.78	52.08
A. fabrum	21.62	30.47	28.32	31.91	29.89	40.21	39.72	44.79	41.61	47.57	49.83	51.56	49.48	55.36	50.52	100	97.92	60.9	61.25	59.03	59.03
***Agrobacterium* ATCC**	21.24	30.11	27.96	31.91	29.18	40.56	40.07	44.44	41.61	47.57	50.17	51.56	49.13	55.02	49.83	97.92	100	60.55	61.25	58.68	57.99
C. cellulolyticum	22.01	26.86	26.5	29.02	26.32	42.66	40.42	44.79	44.98	52.78	49.83	50.34	52.94	58.36	52.25	60.9	60.55	100	94.18	61.94	60.55
***Clostridium* sp.**	22.39	27.3	26.6	29.12	25.35	42.31	41.11	44.79	44.29	53.12	50.87	51.71	53.98	59.25	52.94	61.25	61.25	94.18	100	62.63	61.25
C. scindens	21.71	28.32	25.45	30.5	26.33	39.65	39.51	42.51	41.96	50.87	50.35	51.56	51.74	55.02	52.78	59.03	58.68	61.94	62.63	100	78.2
***Dorea* sp.**	23.26	27.96	26.16	30.5	25.98	38.25	37.06	39.37	41.61	51.22	50.35	52.25	52.43	56.06	52.08	59.03	57.99	60.55	61.25	78.2	100

Fig. S1 Identity (%) matrix of DaeB with the previously characterized ketose 3-epimerases

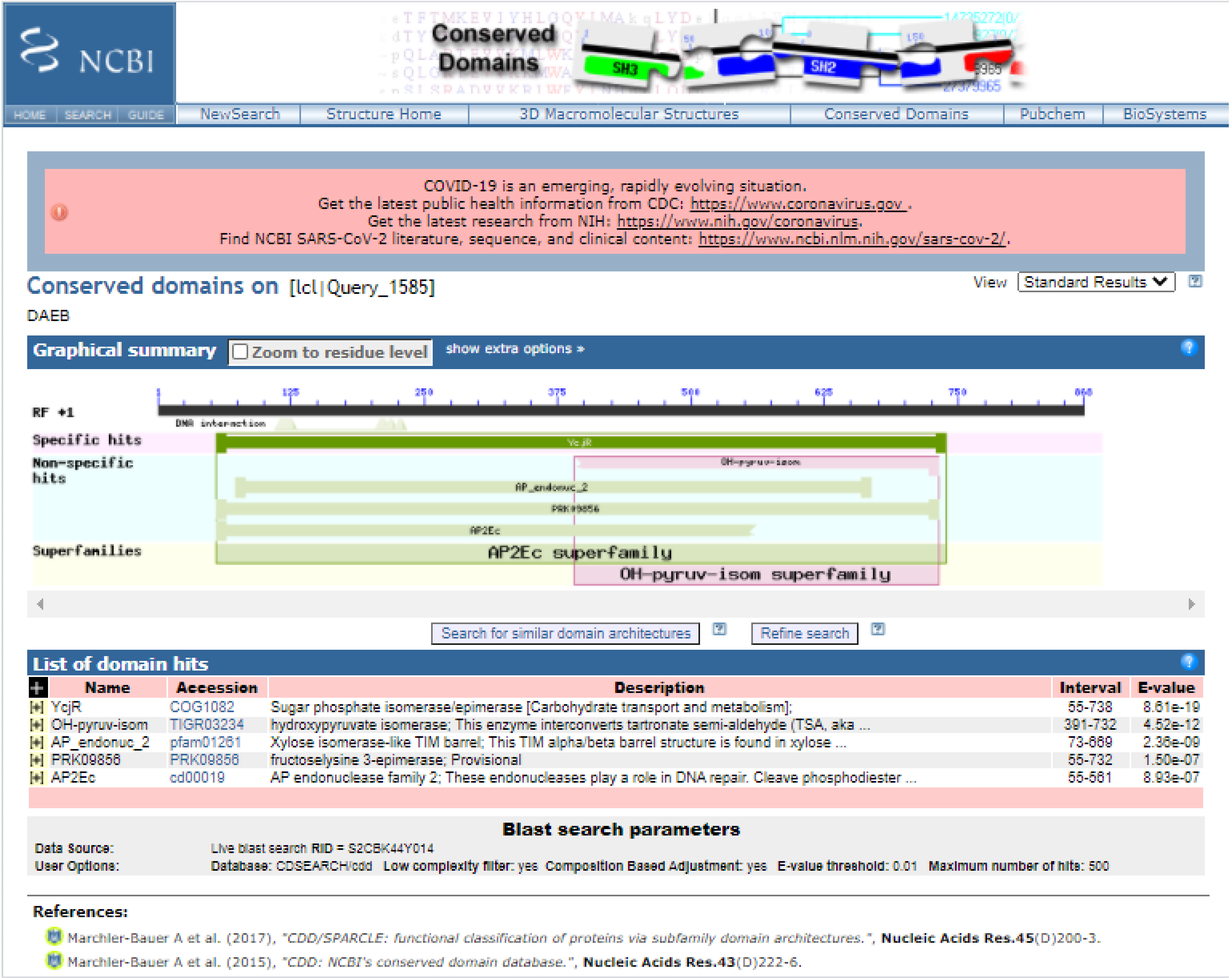

Name	Accession	Description	Interval	E-value
YcjR	COG1082	Sugar phosphate isomerase/epimerase [Carbohydrate transport and metabolism];	55-738	8.61e-19
OH-pyruv-isom	TIGR03234	hydroxypyruvate isomerase; This enzyme interconverts tartronate semi-aldehyde (TSA, aka ...	391-732	4.52e-12
AP_endonuc_2	pfam01261	Xylose isomerase-like TIM barrel; This TIM alpha/beta barrel structure is found in xylose ...	73-669	2.36e-09
PRK09856	PRK09856	fructoselysine 3-epimerase; Provisional	55-732	1.50e-07
AP2Ec	cd00019	AP endonuclease family 2; These endonucleases play a role in DNA repair. Cleave phosphodiester ...	55-561	8.93e-07

Fig. S2 Conserved domain analysis in DaeB protein

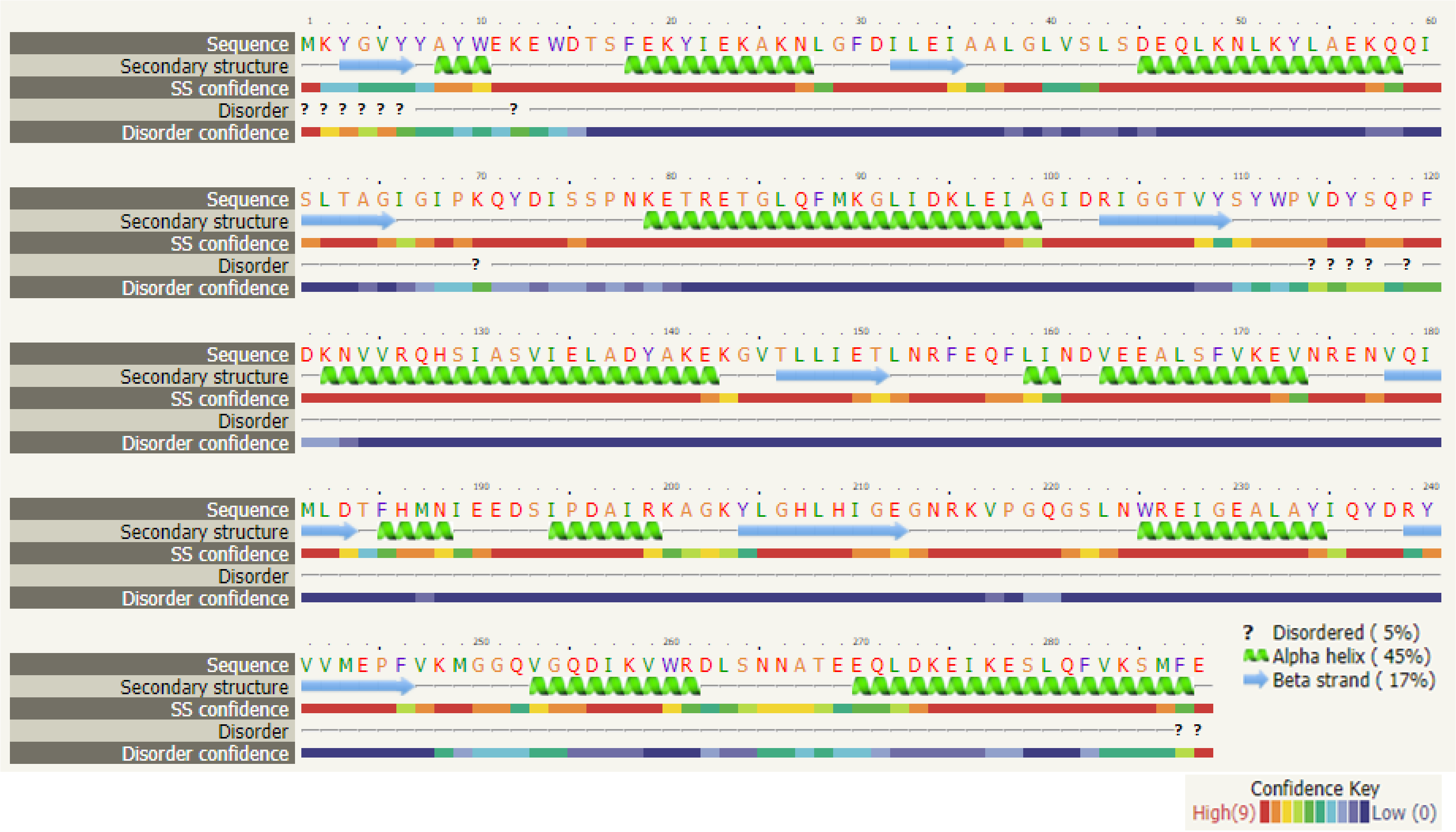

Fig. S3 Predicted secondary structure of DaeB protein sequence. The information has been collected by Phyre2 web based service

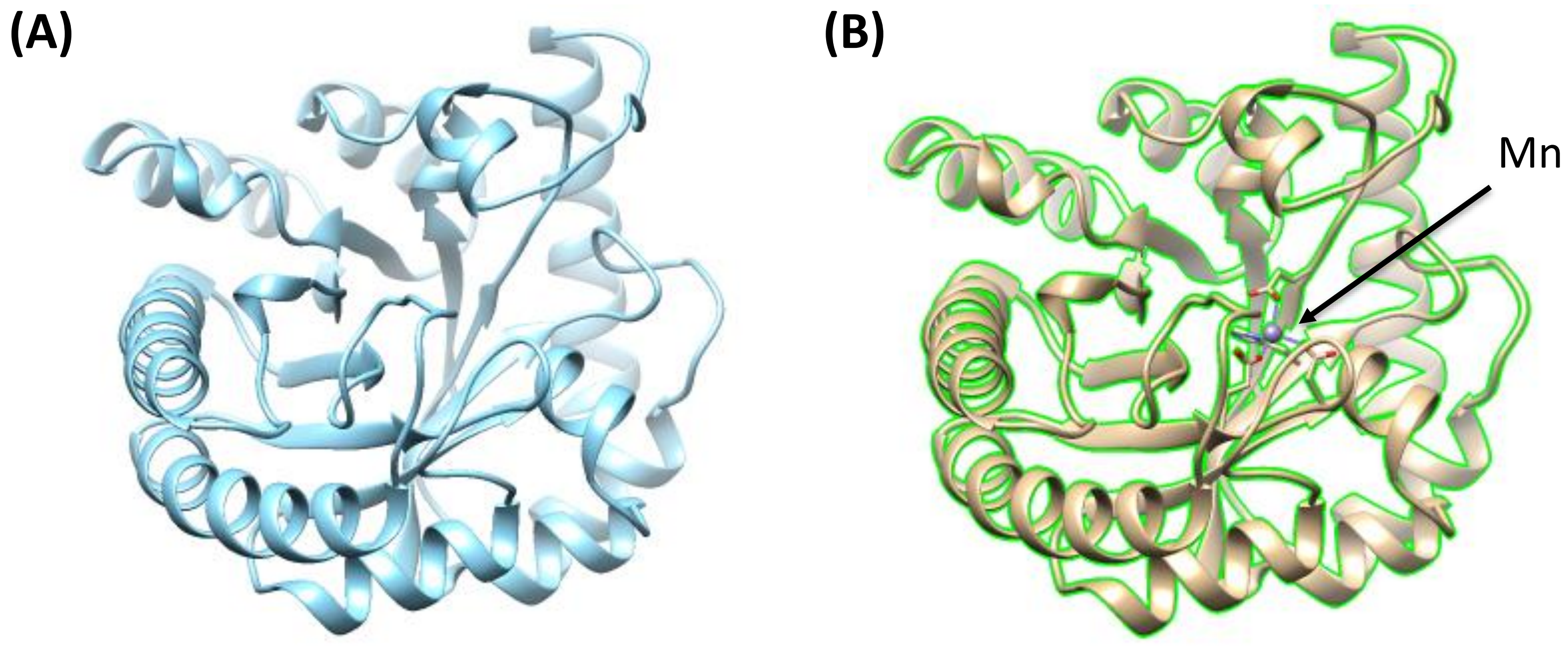

Fig. S4 (A) A predicted 3D homology model of DaeB. A total of 288 amino acid residues of DaeB were modeled at 100% confidence. (B) Superimposition of the homology model of DaeB over the tertiary structure of *C. cellulolyticum* H10 (RMSD value 0.0Å)

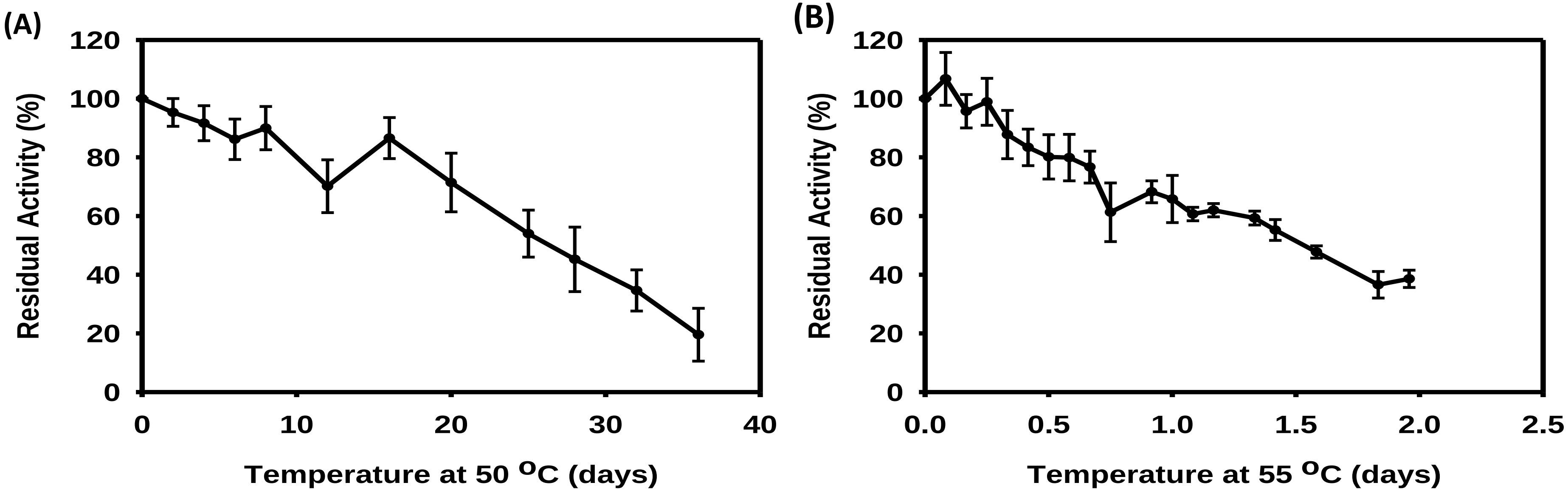

Fig. S5 Effect of temperature on the heat stability of DaeB at pH 8.0

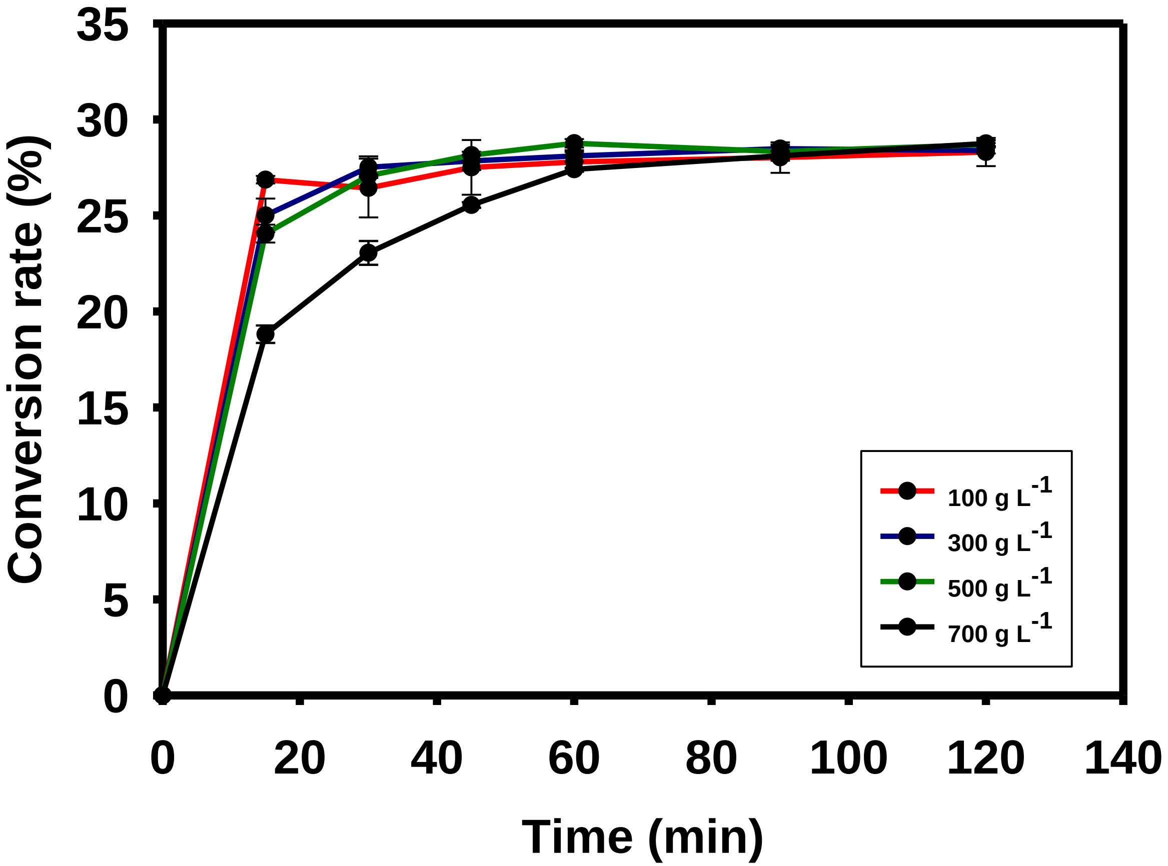

Fig. S6 Time-point conversion of D-allulose from D-fructose (100-700 g L^{-1}) using DaeB enzyme

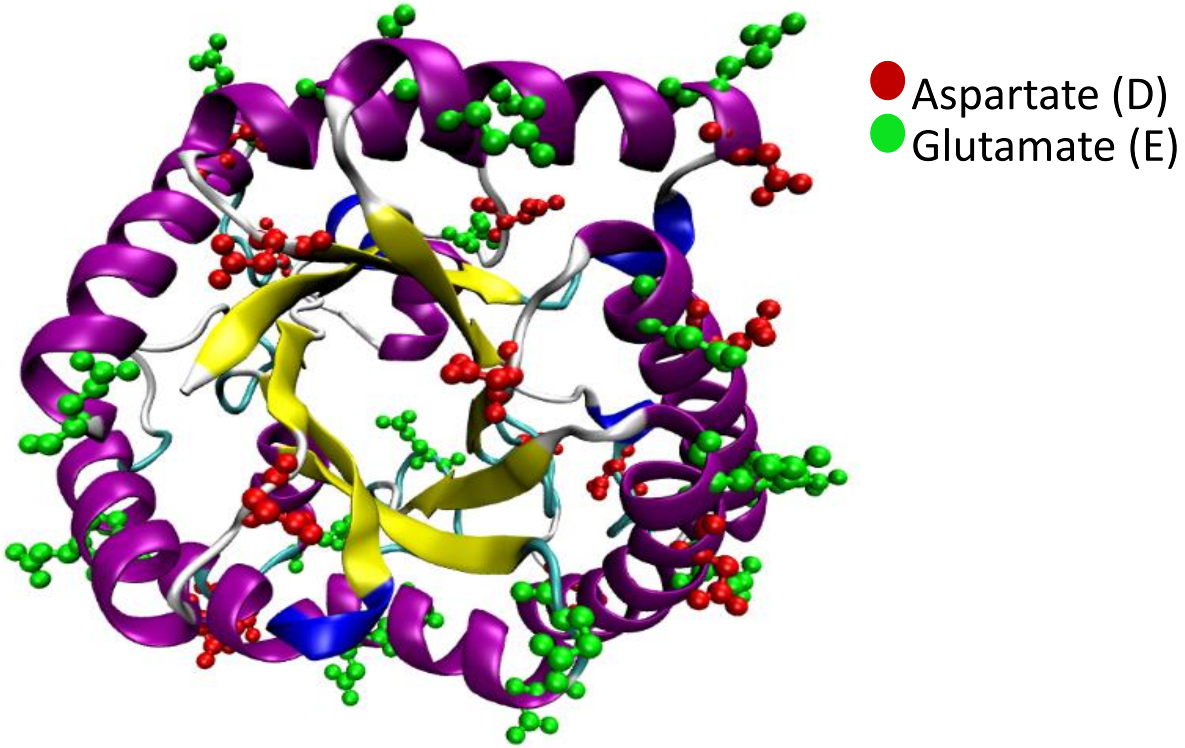

Fig. S7 Homology model of DaeB showing acidic amino acid residues (Asp and Glu) on the surface of the protein

Table S1. Comparison of native molecular mass, kinetic parameters, and specific activity of DaeB and other ketose 3-epimerases.

Enzyme	Source of gene	Native molecular mass	K_m (mM)	k_{cat} (s^{-1})	k_{cat}/K_m (mM^{-1} s^{-1})	Specific activity (U mg^{-1})
D-tagatose 3-epimerase (DTEase)	*Pseudomonas cichorii*	Dimer	NR	NR	NR	NR
	Caballeronia fortuita	Tetramer	81.9	157.21	1.30	270
	Sinorhizobium sp.	Dimer	54.7	50	0.91	NR
D-allulose 3-epimerase (DAEase)	*Agrobacterium tumefaciens*	Tetramer	24	34.46	1.41	8.89
	Clostridium cellulolyticum H10	Tetramer	53.5	55.9	1.045	NR
	Ruminococcus sp.	Tetramer	216	59.4	0.26	8.95
	Clostridium scindens	Tetramer	40.1	5.8	0.145	NR
	Clostridium sp.	Tetramer	279	272	0.97	NR
	Desmospora sp. 8437	Tetramer	549	1059.55	1.93	NR
	Clostridium bolteae	Tetramer	59.8	59	0.99	NR
	Dorea sp. CAG317	Tetramer	153	NR	3.31	803
	Treponema primitia ZAS-1	NR	279	292	1.05	227.5
	Flavonifractor plautii	Tetramer	323	342	1.066	NR
	Arthrobacter globiformis	Tetramer	37.5	41.8	1.115	70.5
	Agrobacterium sp. ATCC 31749	Tetramer	110	106.7	0.98	90.5
	Paenibacillus senegalensis	NR	366.7	40.92	0.11	25.2
	Staphylococcus aureus	Tetramer	59.3	22.9	0.39	NR
	Rhodopirellula baltica	Dimer	78.13	14.90	0.19	11.7
	DaeM	Tetramer	141.43	41.99	0.29	7
	Bacillus sp. KCTC 13219 (DaeB)	Dimer	130	367	2.81	127.2
D-fructose 3-epimerase (DFEase)	*Rhodobacter sphaeroides*	Dimer	NR	NR	NR	NR
L-ribulose 3-epimerase (LREase)	*Mesorhizobium loti*	Tetramer	NR	NR	NR	4.0

Table S2. The conserved active site amino acid residues in the ketose 3-epimerases for which protein structure has been determined, and the homologous residues identified in DaeB.

Active sites	***Bacillus* sp.** KCTC 13219 **(DaeB)**	***Pseudomonas cichorii***	***Agrobacterium tumefaciens***	***Clostridium cellulolyticum***	***Rhodobacter sphaeroides***
**Metal co-coordinating site residue**	Glu150	Glu152	Glu150	Glu150	Glu156
	Asp183	Asp185	Asp183	Asp183	Asp189
	His209	His211	His209	His209	Gln215
	Glu244	Glu246	Glu244	Glu244	Glu250
**Epimerization residues**	Glu150	Glu152	Glu150	Glu150	Glu156
	Glu244	Glu246	Glu244	Glu244	Glu250
**Binding sites for O-1, O-2, and O-3 of the substrate with enzyme**	Glu156	Glu152	Glu156	Glu156	Glu162
	His186	His188	His186	His186	His192
	Asp183			Asp183	
	Arg215	Arg217	Arg215	Arg215	Arg221
	Glu150	Glu152	Glu150	Glu150	Glu156
	Glu244	Glu246	Glu244	Glu244	Glu250
**Binding sites for O-4, O-5, and O-6 of the substrate with enzyme**	Tyr6	Phe7	Tyr6	Tyr6	Ile8
	Trp14	Trp15	Trp14	Trp14	Phe16
	Ile66	Cys66	Ile66	His66	Ala65
	Thr107	Leu108	Ala107	Ala107	Pro107
	Trp112	Trp113	Trp112	Trp112	Arg118
	His209	His211	His209	His209	Gln215
	Phe246	Phe248	Phe246	Phe246	Phe252
**Reference**	This study	[45]	[44]	[46]	[47]

Table S3. *In silco* determination of allergenicity in DaeB protein.

S.No	Allergenicity prediction tools	Allergenic potential
1	Sortaller	Non-allergen
2	AllerTOP	Non-allergen
3	Allermatch	Non-allergen
4	SDAP	Non-allergen

Table S4. Total number of acidic amino acid residues (Asp+Glu), and acidic amino acid residues on the protein's surface of DAEases.

**Source organism of DAEases**	**Total number of acidic amino acid residues**	**Acidic amino acid residues on the protein’s surface**
Clostridium boltea	49	39
Desmospora sp.	49	38
Pseudomonas cichorii	46	38
Bacillus sp. KCTC 13219 (DaeB)	46	37
Agrobacterium fabrum	38	37
Clostridium scindens	47	35
Clostridium cellulolyticum H10	43	35
Flavonifractor plautii	43	35
Dorea sp.	46	34
Rhodopirellula baltica	45	33
Paenibacillus senegalensis	44	33
Caballeronia fortuita	44	33
Rhodobacter sphaeroides	40	33
Sinorhizobium sp.	40	33
Ruminococcus sp.	43	32
Arthrobacter globiformis	41	32
DaeM	42	31
Treponema primitia	41	31
Clostridium sp.	41	31
Staphylococcus aureus	40	31
Agrobacterium ATCC 31749	37	27
